# Keratin/Hydrotalcites Hybrid Sponges as Promising Adsorbents for Cationic and Anionic Dyes

**DOI:** 10.3389/fbioe.2020.00068

**Published:** 2020-02-21

**Authors:** Tamara Posati, Arthur Listwan, Giovanna Sotgiu, Armida Torreggiani, Roberto Zamboni, Annalisa Aluigi

**Affiliations:** ^1^Institute of Organic Synthesis and Photoreactivity, National Research Council, Bologna, Italy; ^2^Chimie Paris Tech – Ecole Nationale Superieure de Chemie de Paris, Paris, France

**Keywords:** keratin, hydrotalcites, sponges, adsorption, water treatment

## Abstract

In this work, keratin sponges were prepared by freeze-drying method and tested for adsorption of Azure A and Methyl Orange dyes. The obtained materials showed a porosity of 99.92% and a mean pore size dimension of about 91 μm. The use of oxidized sucrose with a heating treatment at 150°C was demonstrated to be a useful crosslinking procedure alternative to the conventional glutaraldehyde. Keratin sponges showed a maximum adsorption capacity of 0.063 and of 0.037 mmol/g for Azure A and Methyl Orange, respectively. The absorption of the cationic dye Azure A onto keratin sponges was better described by Freundlich model while the isotherm adsorption of the anionic Methyl Orange was found to correlate with both Langmuir and Freundlich models. The mean free energies evaluated by using the D-R model indicated a physisorption of Methyl Orange and a chemisorptions of Azure A onto keratin sponges. Finally, the functionalization of keratin sponges with Zn Al hydrotalcites nanoparticles did not affect the adsorption performances of the adsorbent toward the cationic dye Azure A, while it improved those toward the anionic Methyl Orange, increasing the related removal efficiencies from 43 to 96%. Collectively, the reported data indicates that the combination of keratin with hydrotalcites nanoparticles is a good strategy to obtain more functional adsorbent materials of potential interest for water treatment and purification.

## Introduction

Today, water pollution is becoming an increasingly serious problem on environment. Particularly, with the increasing applications of aromatic dyes in different industries such as textiles, paper, food, cosmetic, etc., these materials have become dangerous pollutants for aquatic living organism and human health (Aeenjan and Javanbakht, 2018; Afroze and Sen, 2018). Cationic Azure A and anionic Methyl Orange are water soluble aromatic dyes widely used in the textile industry, known to be poisonous and mutagenic (Bulut and Aydin, 2006; Kobya et al., 2006). There are several strategies to treat industrial wastewater, such as biological decomposition, coagulation, ion exchange, adsorption, oxidation processes, etc. Nevertheless, among them, adsorption by means of the use of biomasses, is considered one of the most efficient method to remove pollutant dyes from water, due to its low-cost-biocompatibility and eco-friendliness (Sulyman et al., 2017). As typical biomasses, agricultural products, especially those of polysaccharidic nature, such as chitosan, cellulose or nanocellulose, starch and its derivatives, showed a high potential pollutants uptake, due to their excellent metal-binding and complexing capacities (Lü et al., 2012; Hu et al., 2019; Zhang et al., 2019). However, among biosorbents, keratin wastes are widely considered in recent years as raw materials from which to derive adsorbents for water treatment.

Keratin is the most abundant non-food protein, being the principal component of wool, hair, horns, nails, and feather. Keratin based by-products such as poor quality raw wool (not fit for spinning) deriving from dairy industry, as well as hair and feather from slaughterhouse, account worldwide several million tons per year (Vineis et al., 2019). The disposal of these keratin biomasses represents a problem to be faced because their burning for fuel is inefficient (keratin is self-extinguishing) and polluting (because of the high sulfur content). Moreover, the continuous accumulation in the eco-system gives rise to landscape degradation, as well as pollution of soil and groundwater (Salminen and Rintala, 2002). Keratin protein is characterized by a large number of chemical groups able to complex cationic species, thereby being widely studied as adsorbent for heavy-metal and dyes (Aluigi, 2012; Aluigi et al., 2013). The negative charge of keratin at neutral pH makes the protein highly affine especially for positive ionic species; however, the properly functionalization of keratin based materials could improve the overall adsorption ability also toward anionic molecules. To this purpose, layered double hydroxides (LDHs) or hydrotalcites (HTs) have been recognized as potential adsorbents of anions, due to their anion exchange capacities, low cost and non-toxicity (Gupta et al., 2005; Posati et al., 2014; Martini et al., 2018). HTs comprise a class of layered materials with positively charged layers and charge balancing anions in the interlayer regions (Das et al., 2006). They are generally expressed by the formula [M(II)_1−x_M(III)_x_(OH)_2_]^x+^[${\text{A}}_{\text{x}/\text{n}}^{\text{n}-}$] mH_2_O where M(II) is a divalent cation such as Mg, Ni, Zn, Cu, or Co, M(III) is a trivalent cation such as Al, Cr, Fe, or Ga, and A^n−^ is an anion of charge n. Recently, hydrotalcites have been successfully dispersed in keratin-based matrices as films and nanofibres in order to obtain functional drug release systems (Posati et al., 2018; Giuri et al., 2019). In the present work, the adsorption the aforementioned keratin-based hybrid systems, in form of porous sponges, have been tested as potential adsorbents for both cationic and anionic species. The adsorption tests were performed only on hybrid sponges cross-linked with glutaraldehyde as it represents a standard and well-consolidated crosslinkers agent.

A further drawback of the developed keratin-based materials (such as films, sponges, and nanofibers) is their stability in water. In order to obtain water stable keratin materials an efficient crosslinking is required and glutaraldehyde (GTA) has been widely recognized for its high potency and effectiveness as a crosslinker. However, there are concerns about the GTA use, even at low concentrations (i.e., 0.05% V/V), due to its toxicity (Bigi et al., 2001). In order to replace cytotoxic cross-linkers, the use of saccharides such as sugars has been investigated (Cortesi et al., 1998; Ulubayram et al., 2002). In particular, oxidized sucrose has already showed to be effective in cross-linking zein, gelatin, and feather keratin (Cortesi et al., 1998; Xu et al., 2015b; Mi et al., 2019).

In this work, the adsorption performances of keratin sponges toward Azure A and Methyl Orange were investigated by studying the effect of initial dyes concentration on the adsorption capacity and removal efficiency, as well as sucrose and oxidized sucrose were tested as cross-linkers alternative to glutaraldehyde, even followed by heating treatment. Keratin sponges were prepared by using the freeze-drying methods and characterized in terms of porosity, pore size dimensions, water stability and water uptake. As regards the adsorption mechanism, it was investigated by applying the fitting of the experimental adsorption isotherms with the Langmuir, Freundlich, and Dubinin-Radushkevich model.

In addition, preliminary results about the HTs effect on the adsorption performances of HT/keratin hybrid sponges toward cationic Methylene Blue and anionic Methyl Orange were obtained.

## Experimental Section

### Materials

High molecular weight keratin powder (~50 kDa) extracted from raw wool was kindly donated by Kerline srl. All chemicals were of analytical grade and purchased from Sigma-Aldrich.

### Methods

#### Preparation of Keratin and Keratin/Hydrotalcites Hybrid Sponges

Keratin powder was dissolved in water (10% on weight) containing glycerol (25% on weight vs. keratin) and maintained under stirring for 3 h. After that, the cross-linker was added to the keratin solution (see Table 1). After allowing the crosslinker to act for a suitable time, the solution was placed in the freezer overnight and then freeze-dried in order to obtain the sponges. An half of the sponge samples treated with sucrose and oxidized sucrose were heated at 150°C for 30 min in a fan oven (Table 1). The ZnAl Hydrotalcites (HT) were synthesized by following the method described previously (Bellezza et al., 2009) (see the Supplementary Materials section for a procedure summary). The preparation of the HT loaded keratin sponges was carried out as described before, except that the keratin powder (100 mg) was dissolved in a aqueous suspension (1 mL) of HT (10 mg).

**Table 1:** List of cross-linked keratin sponges.

**Sample**	**Cross-linker**	**Cross-linker amount**	**Cross-linking time (h)**	**Post cross-linking**
KS-GA-24	Glutaraldehyde 25%	0.4 μL per mg of keratin	24	No
KS-S-1	Sucrose	10% vs. keratin	1	No
KS-S-24	Sucrose	10% vs. keratin	24	No
KS-S-1-H	Sucrose	10% vs. keratin	1	Heating
KS-S-24-H	Sucrose	10% vs. keratin	24	Heating
KS-OS-1	Oxidized Sucrose	10% vs. keratin	1	No
KS-OS-24	Oxidized Sucrose	10% vs. keratin	24	No
KS-OS-1-H	Oxidized Sucrose	10% vs. keratin	1	Heating
KS-OS-24-H	Oxidized Sucrose	10% vs. keratin	24	Heating

#### Keratin Sponges Characterization

The morphology of keratin-based sponges was characterized by Scanning Electron Microscopy (SEM) using a Zeiss EVO LS 10 LaB6 scanning electron microscope with an acceleration voltage of 5 kV and a working distance of 5 mm. Samples were gold-sputtered for 1 min before the analysis. Pore size was measured using GIMP 2.8 as software image. In particular, the pore size mean dimension and its standard deviation were obtained from 50 measurements, randomly gathered from several SEM photos of two different samples. The density (ρ) was determined by using the mass of the samples in air (*m*_1_) and in dodecane (*m*_2_) through the following Equation 1:

(1) $$\begin{array}{r} \rho =\left(m_{1}\rho_{h}\right)/\left(m_{1}-m_{2}\right) \end{array}$$

where

ρ_*h*_ (g/cm^3^) is the density of the dodecane at 25°C. The porosity (ε) was then determined by using the Equation 2:

(2) $$\begin{array}{r} \varepsilon =\frac{V_{TOT}-V}{V_{TOT}}\times 100 \end{array}$$

where *V*_*TOT*_ is the total (apparent) volume, obtaining by measuring the volume of the cylindrical sponge. In order to evaluate the stability of the cross-linked sponges, the dried samples were weighted (*m*_1_), then soaked in water for 24 h (about 13 mg of sample for mL of water), under room temperature. Then, the water was removed and the sponges were freeze-dried again (*m*_2_). The mass loss (%) was calculated by applying the following Equation 3:

(3) $$\begin{array}{r} Mass\;loss\;\left(\%\right)=\frac{m_{1}-m_{2}}{m_{1}}\times 100 \end{array}$$

For the water uptake test, the freeze-dried sponges were weighted (*w*_*dry*_), soaked in a vial containing water (about 13 mg of sample for mL of water) for 24 h at room temperature and weighted again in the wet state (*w*_*wet*_). The water uptake was calculated according to the following Equation 4:

(4) $$\begin{array}{r} Water\;uptake\;\left(\%\right)=\frac{w_{wet}-w_{dry}}{w_{dry}}\times 100 \end{array}$$

#### Adsorption Test

For the adsorption tests, the dyes stock solutions of AzureA (AzA) and Methyl Orange (MO) at a concentration of 1,000 mg/L were prepared for performing the adsorption tests. The initial concentrations of the experimental solutions were obtained by properly diluting the dyes stock solutions in a 50–500 mg/L concentration range. The adsorption test were carried out in batch conditions by immersing the keratin sponges (about 23 mg) in 1 mL of starting dye solution, at neutral pH and room temperature for 24 h. The initial and final dyes concentration was determined by using a spectrophotometer Cary 100 (Agilent Technologies), at the wavelengths of 630 and 470 nm, for AzA and MO, respectively. The adsorption capacities *Q*(*mmolg*) and the removal efficiency *R*(%) were evaluated using the Equation 5 and 6, respectively:

(5) $$Q\left(\frac{mmol}{L}\right)\;=\;\frac{(C_{0}-C_{f})\times V}{m}$$

(6) $$\begin{array}{r} R\left(\%\right)=\frac{C_{0}-C_{f}}{C_{0}}\times 100 \end{array}$$

where $C_{0}\left(\frac{mmol}{L}\right)$ and $C_{f}\left(\frac{mmol}{L}\right)$ are the initial and final dyes concentration, respectively, *V*(*L*) is the volume of dye solutions and *m*(*g*) is the mass of the keratin sponges. The adsorption on keratin sponges loaded with HT (KS-HT) were carried out by using starting solutions at initial dye concentration of 0.76 mmol/L. The KS-HT sponges were initially immersed in the AzA solution for 24 h, and then in the MO solution for further 24 h (KS-HT_*AzA*→*MO*_). The opposite procedure was also followed to verify eventual differences (sponges first immersed in MO and then in AzA solution—KS-HT_*MO*→*AzA*_). The adsorption capacities and removal efficiencies of the dyes were evaluated as described before.

## Results and Discussion

### Effect of Crosslinking Methods on Water Stability and Water Uptake of the Keratin Sponges

**Figure 1:**
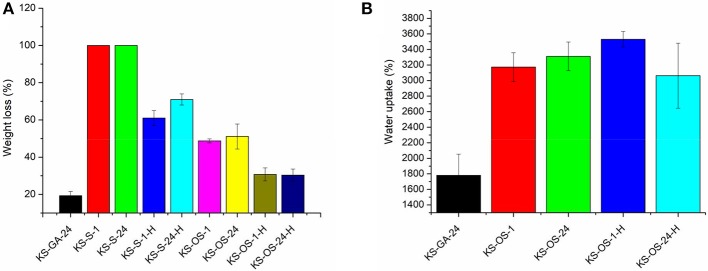
Water stability **(A)** and swelling ability **(B)** of keratin sponges crosslinked with glutaraldehdye (KS-GA-24), with sucrose for 1 h (KS-S-1) and for 24 h (KS-S-24), even followed by heating treatment (KS-S-1-H and KS-S-24H, repsecitvely) and with oxidized sucrose for 1 h (KS-OS-1) and 24 h (KS-OS-24H), even followed by heating treatment (KS-OS-1-H and KS-OS-24-H, respectively).

Freeze-dried keratin sponges are completely soluble in water, therefore they must be suitably crosslinked for applications in water treatment. Sucrose and oxidized sucrose have been studied as crosslinkers alternative to well-consolidated GTA. The tests with sucrose were carried out in order to verify if the Maillard reaction, which occurs between protein and carbohydrate at about 180°C, synergistically contributes to the sponges crosslinking. Several cross-linking procedures were tested by changing different factors such as the cross-linker compound, the cross-linking time, and an eventual post-treatment by heating (Table 1). The synthesis of oxidized sucrose, as well as the crosslinking reactions among keratin and all the crosslinkers taken in consideration in this work, are described in the Supplementary Materials. As shown in Figure 1A, the sponges treated only with sucrose, totally dissolved after being in water for 24 h (samples KS-S-1, KS-S-24). However, with the post-cross-linking by heating, the weight loss in water reduced to 61% sucrose action for 1 h (KS-S-1-H) and to 71% when it is left to act for 24 h (KS-S-24-H). Based on these results, there is no evidence of chemical reaction between keratin and sucrose at room temperature. The slight increased stability observed in the sucrose-treated and heated samples can be attributed to the formation of internal crosslinking between acid and amino groups of the protein (Aluigi et al., 2013). On the other hand, keratin sponges cross-linked with oxidized sucrose (OS) for 1 and 24 h (KS-OS-1 and KS-OS-24), showed weight loss of 49 and 51%, respectively. For both samples, the weight loss is reduced to about 30% with the post heating treatment (KS-OS-1-H and KS-OS-24-H), thereby becoming comparable with that of GTA (about 20%). This result indicates that the cross-linking reaction between keratin and oxidized sucrose is not time-dependent at room temperature, while it needs the removal of water molecules by heating to be completed (Xu et al., 2015a). In order to measure the ability of the cross-linked sponges to retain water, the swelling tests were carried out (Figure 1B). Compared to GTA crosslinked sponges, the results showed a significant increase in water uptake ability of OS crosslinked keratin sponges. This is probably due to higher ability of OS compared to GTA, to links water molecules by means of hydrogen bonds with its hydroxyl groups (Figures S3, S4).

### Morphology of Keratin Sponges Used for the Adsorption Test

**Figure 2:**
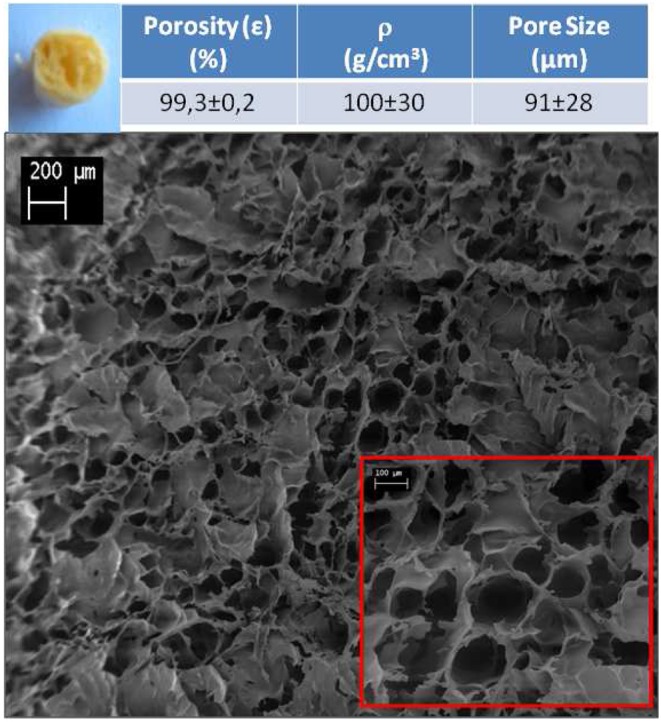
Optical appearance and SEM image of the section of keratin sponges.

The dyes adsorption tests were carried out on the GTA cross-linked sponges having a diameter of 0.8 cm and a thickness of 0.7 cm. In Figure 2, the photograph and the SEM images of the sponge section are reported. The sample showed a 3D-porous structures made of open pores visually interconnected. Moreover, the sponge showed a high porosity of about 99% and a pore size dimension of about 91 ± 28 μm. As concern the keratin/HT hybrid sponges, their SEM images revealed that hydrotalcites are homogeneously dispersed in the keratin matrix (Figure S5).

### Effect of Initial Dyes Concentration on Adsorption

**Figure 3:**
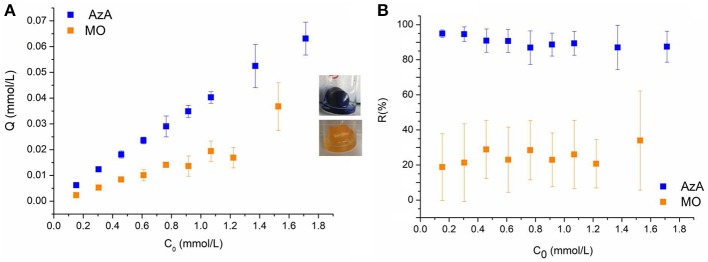
**(A)** Adsorption capacity Q (mmol/L) and **(B)** Removal Efficiency R (%) as functions of initial dyes concentration.

The effect of the initial dyes concentration on the adsorption capacity and removal efficiency of the keratin sponge is shown in Figure 3. As expected, for both dyes, the adsorption capacity increased with increasing the initial dyes concentration. This is due to the mass transfer increase between the aqueous and solid phase with the increasing of the initial dyes concentration (Aluigi et al., 2014). On the other hand, the removal efficiency slightly decreased with increasing the initial dyes concentration. In particular, by increasing the initial dyes concentration from 0.15 to 1.8 mmol/L the adsorption capacity increased from 0.00061 to 0.063 mmol/g for AzA and from 0.0023 to 0.037 mmol/g for MO; conversely, the removal efficiency decreased from 94 to 87% for AzA and from 56 to 35% for MO. As expected the keratin sponge showed a higher affinity toward the AzA than MO. This is due to the to the overall negative charge that the protein assumes at pH higher than its isoelectric point (4.0–4.5), which promotes the electrostatic interactions with cationic molecules as AzA.

### Adsorption Isotherm Study

The adsorption processes of AzA and MO on the keratin sponge were studied by fitting the experimental adsorption values with different isotherm models, namely, Langmuir, Freundlich, and Dubinin-Radushkevich models. The Langmuir model (Equation 7) describes a mono layer adsorption process assuming that the adsorption occurs at specific homogenous sites within the adsorbent (Wang et al., 2005):

(7) $$\begin{array}{r} Q_{e}=Q_{m}K_{L}\frac{C_{e}}{1+K_{L}C_{e}} \end{array}$$

where *Q*_*e*_ (mmol/g) is the adsorption capacity at the equilibrium, *Q*_*m*_ (mmol/g) is the maximum adsorption capacity, *C*_*e*_ (mmol/L) is the equilibrium concentration of the dyes in solutions and *K*_*L*_ (L/mmol) is the effective dissociation constant. By using the Langmuir model it is possible to calculate the separation factor *R*_*L*_ (Equation 8) which indicates whether an adsorption system is irreversible (*R*_*L*_ = 0), favorable (0 < *R*_*L*_ < 1) or unfavorable (*R*_*L*_> 1):

(8) $$\begin{array}{r} R_{L}=\frac{1}{1+K_{L}C_{0}} \end{array}$$

The Freundlich model is commonly used to describe the adsorption process on the heterogeneous surface and it is applicable when the amount of adsorbed solute increases indefinitely with the concentration of solute in the starting solution (Dubey and Gopal, 2007). The Freundlich isotherm is described by the following Equation:

(9) $$\begin{array}{r} Q_{e}=K_{f}C_{e}^{\frac{1}{n}} \end{array}$$

where *K*_*f*_ (mmol/g) (L/mmol)^1/n^ is a constant indicative of the adsorption capacity and *n* is an empirical constant related to the magnitude of the adsorption driving force. An *n* value between 1 and 10 indicates a favorable adsorption.

Finally, the Dubinin-Radushkevich (D-R) model is generally applied to determine the adsorption mechanism. This model is described by the following Equation 10:

(10) $$\begin{array}{r} Q_{e}=Q_{S}e^{\left(-K_{DR}\varepsilon^{2}\right)} \end{array}$$

The parameter *K*_*DR*_ (mol^2^/kJ^2^) is a constant related to the adsorption energy and ε is the Polany potential that can be calculated through the Equation 11:

(11) $$\begin{array}{r} \varepsilon =RTln\left(1+\frac{1}{C_{e}}\right) \end{array}$$

where *R* is the gas constant (kJ/mol) and *T* the temperature (K). The mean free energy *E*(kJ/mol) can be obtained by using the constant *K*_*DR*_ by means of the following Equation 12:

(12) $$\begin{array}{r} E={\left(2K_{DR}\right)}^{-0.5} \end{array}$$

**Figure 4:**
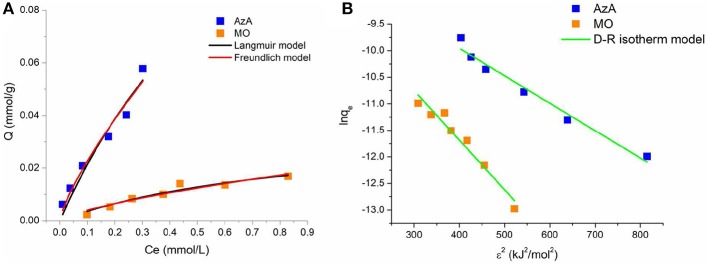
Plots of the fitting of the experimental data with Langmuir and Freundlich **(A)**, and Dubinin-Radushkevich **(B)** isotherm models.

**Figure 5:**
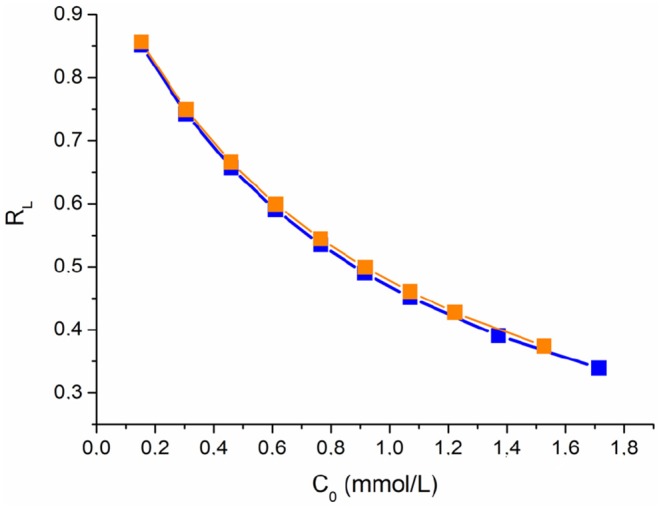
Separation factor for the adsorption of AzA and MO on keratin sponges.

This approach was usually applied to distinguish the physical and chemical adsorption of adsorbate molecules with its mean free energy; indeed the *E* value ranges from 1 to 8 kJ/mol for physical adsorption and from 8 to 16 kJ/mol for chemical adsorption (Saltali et al., 2007; Zheng et al., 2008). The parameters of the applied isotherm models with related correlation coefficients are summarized in Table 2. For the AzA adsorption, the Freundlich model fit the experimental data better than the Langmuir (higher *R*^2^ value and significative parameter values). Instead, the adsorption of MO is described in a satisfactory manner by both Langmuir and Freundlich models (Figure 4A). Moreover, in the selected range of the initial concentration, the *R*_*L*_ values deriving from the Langmuir model fall between 0 and 1 for both dyes, as well as the *n* value of the Freundlich model that is higher than 1, thereby indicating a favorable adsorption (Figure 5).

**Table 2:** Parameters for the AzA and MO adsorption on keratin sponges.

**Model**	**Langmuir**			**Freundlich**			**D-R**		
**Adsorbate**	**Q_*m*_ (mmol/g)**	***K*_*L*_ (L/mmol)**	**R^**2**^**	**K_*f*_**	***n***	**R^**2**^**	**Q_*max*_ (mol/g)**	***E* (kJ/mol)**	**R^**2**^**
AzA	0.2 ± 0.2	1 ± 1	0.94	0.13 ± 0.02	1.3 ± 0.2	0.96	(3.7 ± 0.7) 10^−5^	9.9 ± 0.4	0.97
MO	0.04 ± 0.01	1.1 ± 0.5	0.93	0.020 ± 0.002	1.4 ± 0.2	0.91	(4 ± 1) 10^−5^	7.3 ± 0.3	0.95

Finally, the adsorption type of the dyes onto the keratin sponge was determined by fitting the experimental data with the D-R model (Figure 4B). The mean free energies calculated by using the *K*_*DR*_ values are of 9.9 and 7.3 for AzA and MO, respectively indicating a chemisorption for the cationic dye and a physisorption for the anionic one.

### Effect of HT on the AzA and MO Adsorption Process

**Figure 6:**
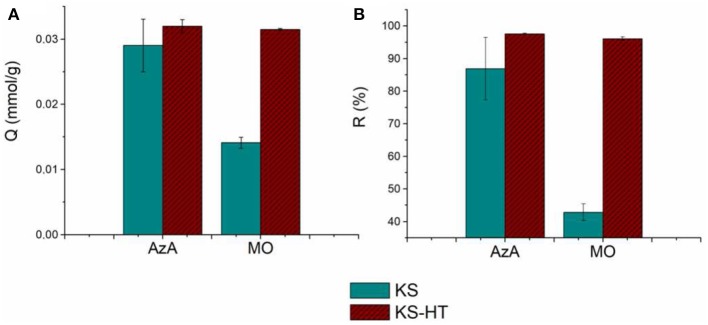
Adsorption capacities **(A)** and Removal Efficiency **(B)** of KS and KS-HT sponges.

**Figure 7:**
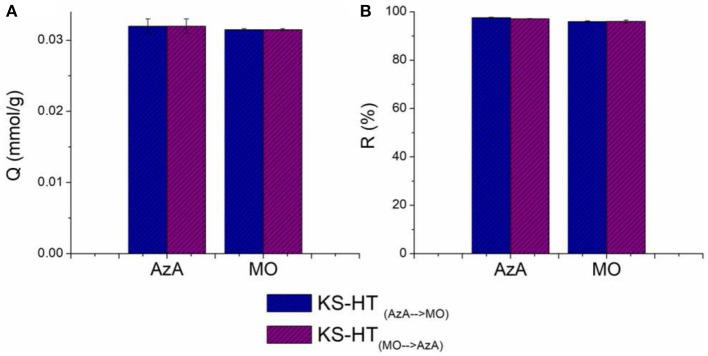
Adsorption capacities **(A)** and Removal Efficiency **(B)** of KS-HT sponges toward first AzA and then MO and *vice versa*.

In order to study the effect of HT nanoparticles on the adsorption processes of the considered organic dye, the adsorption capacities and removal efficiencies of keratin sponge (KS) evaluated at a given concentration of the dyes (0.76 mmol/L) were compared with those of the keratin sponge loaded with 10% on weight of hydrotalcites (KS-HT). In Figure 6 the adsorption capacity and the removal efficiency of the KS-HT sponge toward AzA and MO are shown. No significant changes were observed among KS and KS-HT for the AzA adsorption; therefore the cationic character of HT seems to not interfere with the adsorption capacity of anionic keratin toward AzA. On the other hand the adsorption capacity and removal efficiency of the KS-HT sponge for MO are significant higher than those of KS (adsorption capacity, 0.031 mmol/g instead 0.014 mmol/g and removal efficiencies 96% instead 43%). This is due to the well-known capability of HT to well interact with anionic species. Furthermore, the ability of the same KS-HT to absorb both dyes was also studied. For this type of investigation, we decided not to use a solution containing both dyes, due to their opposite charge, but to perform adsorption test in sequence using the same sponge to absorb first a dye and then the other. Two kinds of adsorption tests were carried out with the swapped order of dyes. The obtained results, shown in Figure 7, revealed that the order in which the sponges are in contact with dyes does not affect their adsorption performances, thereby suggesting that keratin and HT react independently with AzA and MO.

## Conclusion

Keratin porous sponges having a porosity of 99.92% and a mean pore size dimension of 91 μm, were prepared by freeze-drying method and tested for the adsorption of AzA and MO, chosen as models of cationic and anionic dyes, respectively. The use of oxidized sucrose following to the heating treatment at 150°C seems to be a useful crosslinking procedure alternative to glutaraldehyde.

In the considered range of initial dyes concentration, the prepared keratin sponges showed a maximum adsorption capacity of 0.063 and of 0.037 mmol/g for AzA and MO, respectively. Among the Langmuir and Freundlich models applied to the adsorption data, the Freundlich was the best model to describe the adsorption of AzA; while, the isotherm adsorption of MO was found to correlate with both Langmuir and Freundlich models. The mean free energies evaluated by using the D-R model resulted of 9.9 kJ/mol for AzA and of 7.3 for MO. This indicates a pysisorption of MO and a chemisorption of AzA onto keratin sponges, which implies electrostatic interactions between the cationic dye and the protein.

Finally, the functionalization of keratin sponges with HT does not affect the adsorption performances of the adsorbent toward AzA, while it improve those toward MO, by increasing the related adsorption capacity from 0.014 to 0.031 mmol/g and the related removal efficiencies from 43 to 96%. This evidenced that the hydrotalcites improve the adsorption performances of the keratin-based materials, only toward the anionic dye. Moreover, tests performed to study the adsorption performances of the same keratin/HT sponge toward AzA and MO indicated that keratin and HT act toward the cationic and the anionic dye, respectively, in a independent manner, without interfering each other. These preliminary test carried out on the keratin/HT sponge revealed that the idea to combine keratin with hydrotalcites appear successful to obtain a more functional adsorbent. Therefore, a detailed study of the adsorption mechanisms of AzA and MO on the keratin sponge with hydrotalcites, as well as the simultaneous and the selective adsorption of the two types of dyes will be the object of the further work.

## Data Availability Statement

All datasets generated for this study are included in the article/Supplementary Material.

## Author Contributions

This work is a result of the internship period at the CNR-ISOF of the student AL from the Chimie ParisTech. AA was supervisor of the Erasmus Student AL, followed the student in the preparation and characterization of the keratin sponges by freeze-drying, worked on the study design and writing, coordinated the data collection and discussion for the realization of this work, and also dealt with the mathematical model elaboration of adsorption experimental data. TP has given a great support in the work design and writing, followed the student in the synthesis of the hydrotalcites and in the chemical-physical characterization of the keratin sponges and contributed to the data discussion and paper revision. AL within the Erasmus project, worked under the supervision of AA in preparing the samples and collecting the laboratory experimental data. GS was involved in the synthesis of oxidized sucrose and in the experimental work related to the experimental determination of dyes adsorption curves by UV-Vis spectrophotometric measurements and contributed to the data discussion and paper revision. AT was the leader of the RM @ School project, which funded the publication of this work, was involved in the characterization of cross-linkers and keratin-based materials, gave a great support for the FTIR characterizations, and contributed to the data discussion and paper revision. RZ was the director of the ISOF (Institute for the Organic Synthesis and Photoreactivity) and contributed to the data discussion and to the paper revision.

### Conflict of Interest

The authors declare that the research was conducted in the absence of any commercial or financial relationships that could be construed as a potential conflict of interest.
