# Supplementary material for: Keratin/Hydrotalcites Hybrid Sponges as Promising Adsorbents for Cationic and Anionic Dyes
Source: Front Bioeng Biotechnol. 2020 Feb 21;8:68. doi: 10.3389/fbioe.2020.00068 (PMC7047220; doi:10.3389/fbioe.2020.00068)
Supplement: Supplementary file 1 [file Presentation_1.PPTX]

## Slide 1
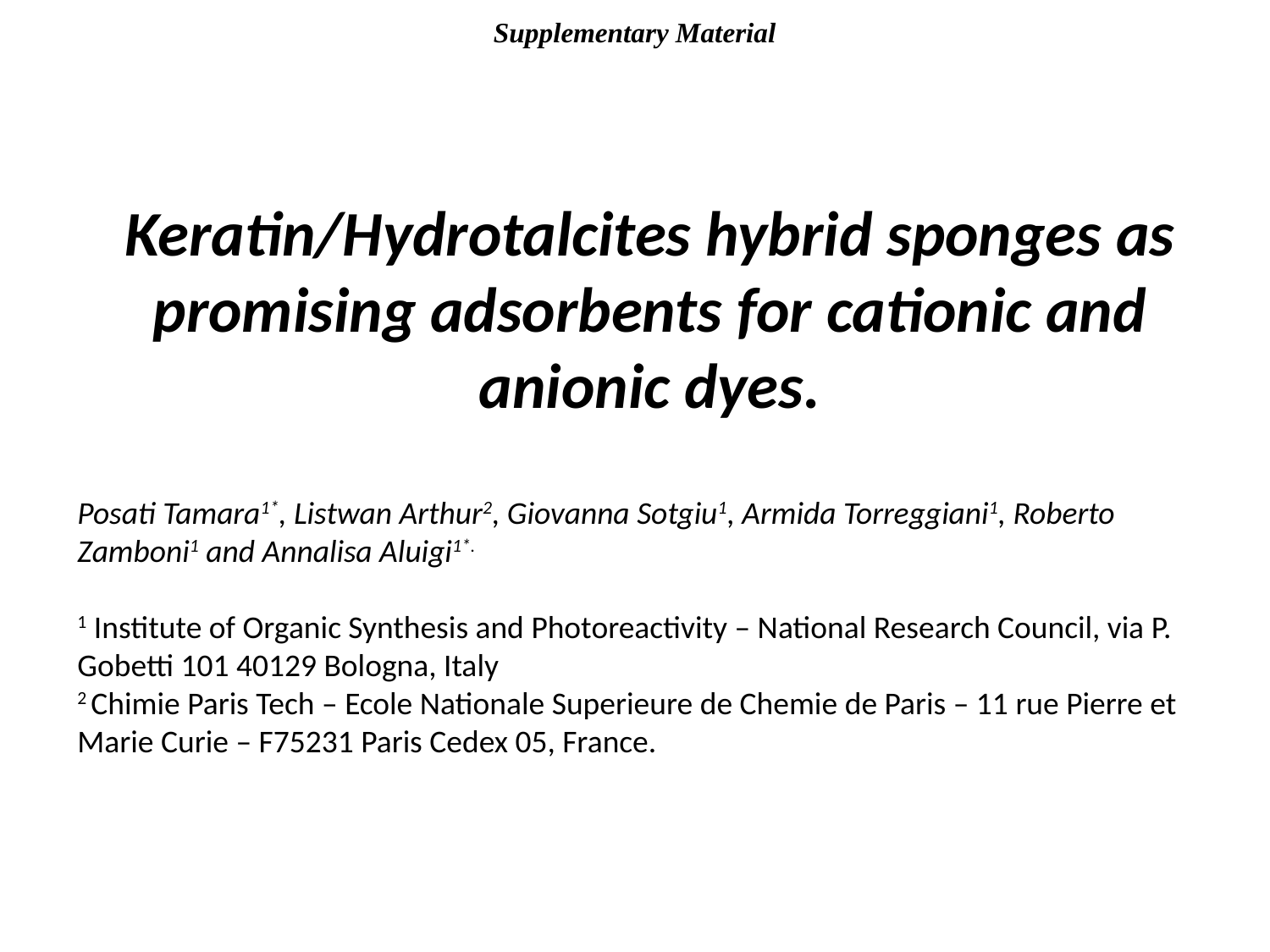

Supplementary Material
Keratin/Hydrotalcites hybrid sponges as promising adsorbents for cationic and anionic dyes.
Posati Tamara1*, Listwan Arthur2, Giovanna Sotgiu1, Armida Torreggiani1, Roberto Zamboni1 and Annalisa Aluigi1*.
1 Institute of Organic Synthesis and Photoreactivity – National Research Council, via P. Gobetti 101 40129 Bologna, Italy
2 Chimie Paris Tech – Ecole Nationale Superieure de Chemie de Paris – 11 rue Pierre et Marie Curie – F75231 Paris Cedex 05, France.

## Slide 2
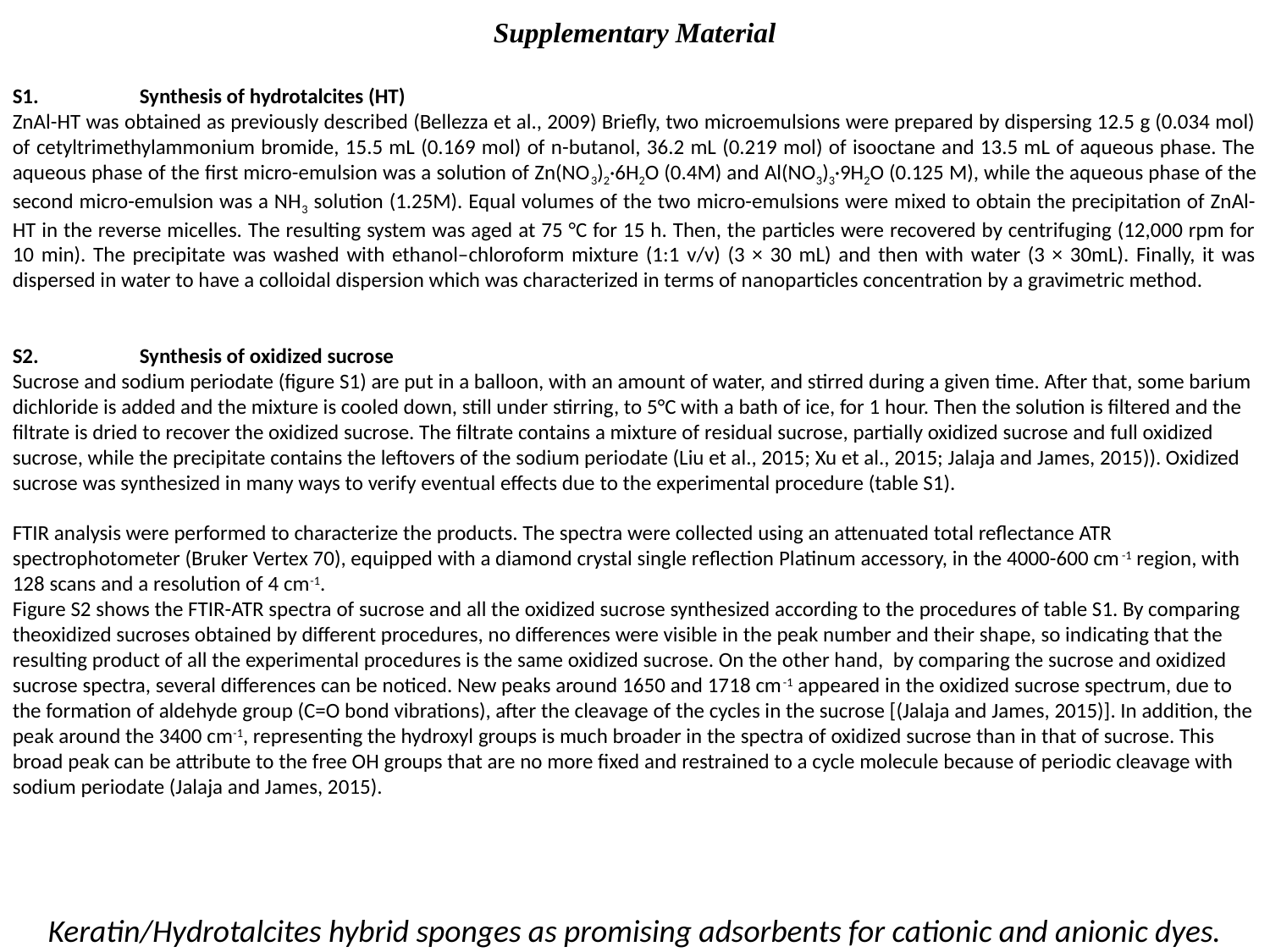

Supplementary Material
S1.	Synthesis of hydrotalcites (HT)
ZnAl-HT was obtained as previously described (Bellezza et al., 2009) Briefly, two microemulsions were prepared by dispersing 12.5 g (0.034 mol) of cetyltrimethylammonium bromide, 15.5 mL (0.169 mol) of n-butanol, 36.2 mL (0.219 mol) of isooctane and 13.5 mL of aqueous phase. The aqueous phase of the first micro-emulsion was a solution of Zn(NO3)2·6H2O (0.4M) and Al(NO3)3·9H2O (0.125 M), while the aqueous phase of the second micro-emulsion was a NH3 solution (1.25M). Equal volumes of the two micro-emulsions were mixed to obtain the precipitation of ZnAl-HT in the reverse micelles. The resulting system was aged at 75 °C for 15 h. Then, the particles were recovered by centrifuging (12,000 rpm for 10 min). The precipitate was washed with ethanol–chloroform mixture (1:1 v/v) (3 × 30 mL) and then with water (3 × 30mL). Finally, it was dispersed in water to have a colloidal dispersion which was characterized in terms of nanoparticles concentration by a gravimetric method.
S2.	Synthesis of oxidized sucrose
Sucrose and sodium periodate (figure S1) are put in a balloon, with an amount of water, and stirred during a given time. After that, some barium dichloride is added and the mixture is cooled down, still under stirring, to 5°C with a bath of ice, for 1 hour. Then the solution is filtered and the filtrate is dried to recover the oxidized sucrose. The filtrate contains a mixture of residual sucrose, partially oxidized sucrose and full oxidized sucrose, while the precipitate contains the leftovers of the sodium periodate (Liu et al., 2015; Xu et al., 2015; Jalaja and James, 2015)). Oxidized sucrose was synthesized in many ways to verify eventual effects due to the experimental procedure (table S1).
FTIR analysis were performed to characterize the products. The spectra were collected using an attenuated total reflectance ATR spectrophotometer (Bruker Vertex 70), equipped with a diamond crystal single reflection Platinum accessory, in the 4000-600 cm-1 region, with 128 scans and a resolution of 4 cm-1.
Figure S2 shows the FTIR-ATR spectra of sucrose and all the oxidized sucrose synthesized according to the procedures of table S1. By comparing theoxidized sucroses obtained by different procedures, no differences were visible in the peak number and their shape, so indicating that the resulting product of all the experimental procedures is the same oxidized sucrose. On the other hand, by comparing the sucrose and oxidized sucrose spectra, several differences can be noticed. New peaks around 1650 and 1718 cm-1 appeared in the oxidized sucrose spectrum, due to the formation of aldehyde group (C=O bond vibrations), after the cleavage of the cycles in the sucrose [(Jalaja and James, 2015)]. In addition, the peak around the 3400 cm-1, representing the hydroxyl groups is much broader in the spectra of oxidized sucrose than in that of sucrose. This broad peak can be attribute to the free OH groups that are no more fixed and restrained to a cycle molecule because of periodic cleavage with sodium periodate (Jalaja and James, 2015).
Keratin/Hydrotalcites hybrid sponges as promising adsorbents for cationic and anionic dyes.

## Slide 3
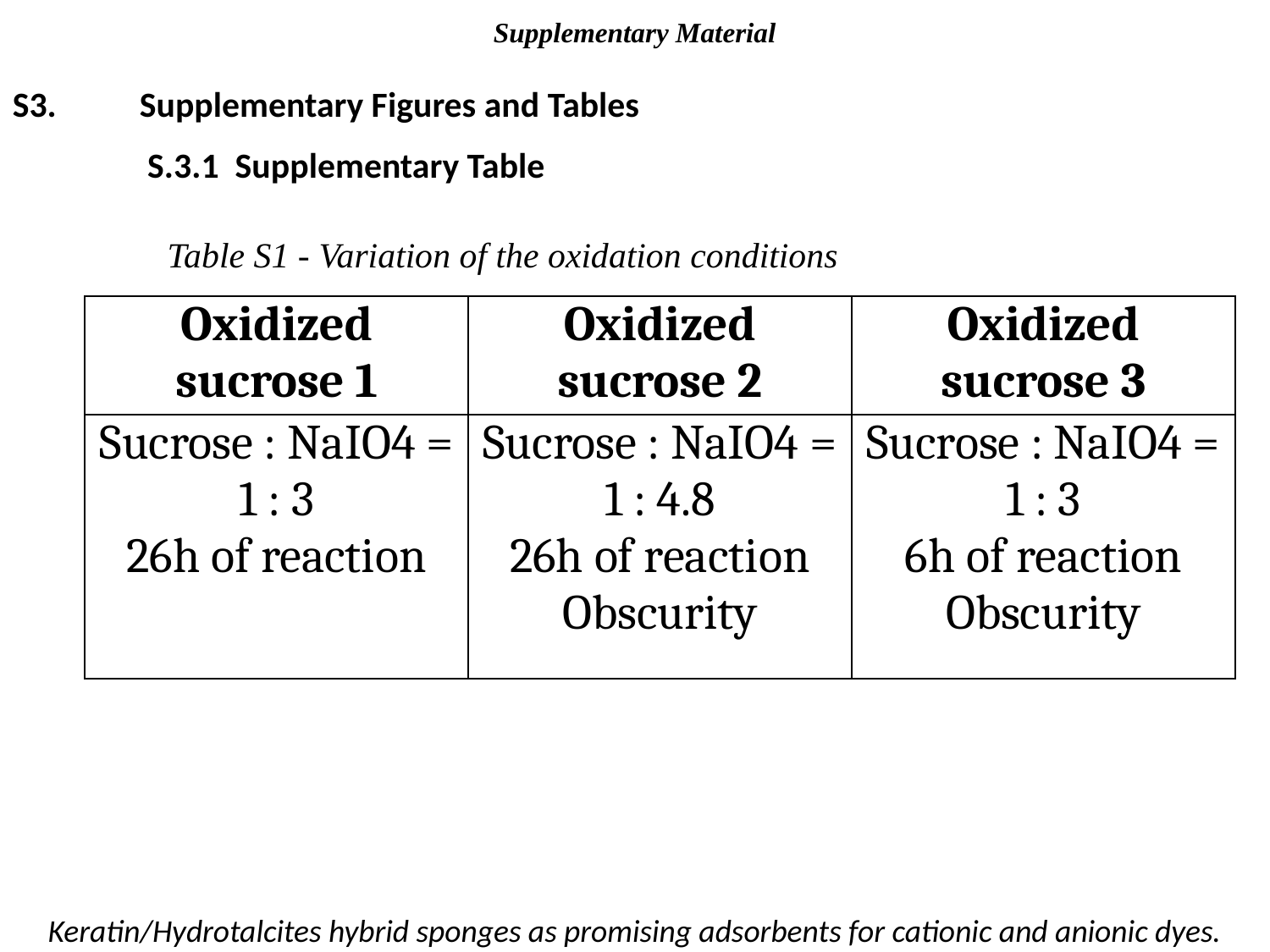

Supplementary Material
S3.	Supplementary Figures and Tables
S.3.1 Supplementary Table
Table S1 - Variation of the oxidation conditions
| Oxidized sucrose 1 | Oxidized sucrose 2 | Oxidized sucrose 3 |
| --- | --- | --- |
| Sucrose : NaIO4 = 1 : 3 26h of reaction | Sucrose : NaIO4 = 1 : 4.8 26h of reaction Obscurity | Sucrose : NaIO4 = 1 : 3 6h of reaction Obscurity |
Keratin/Hydrotalcites hybrid sponges as promising adsorbents for cationic and anionic dyes.

## Slide 4
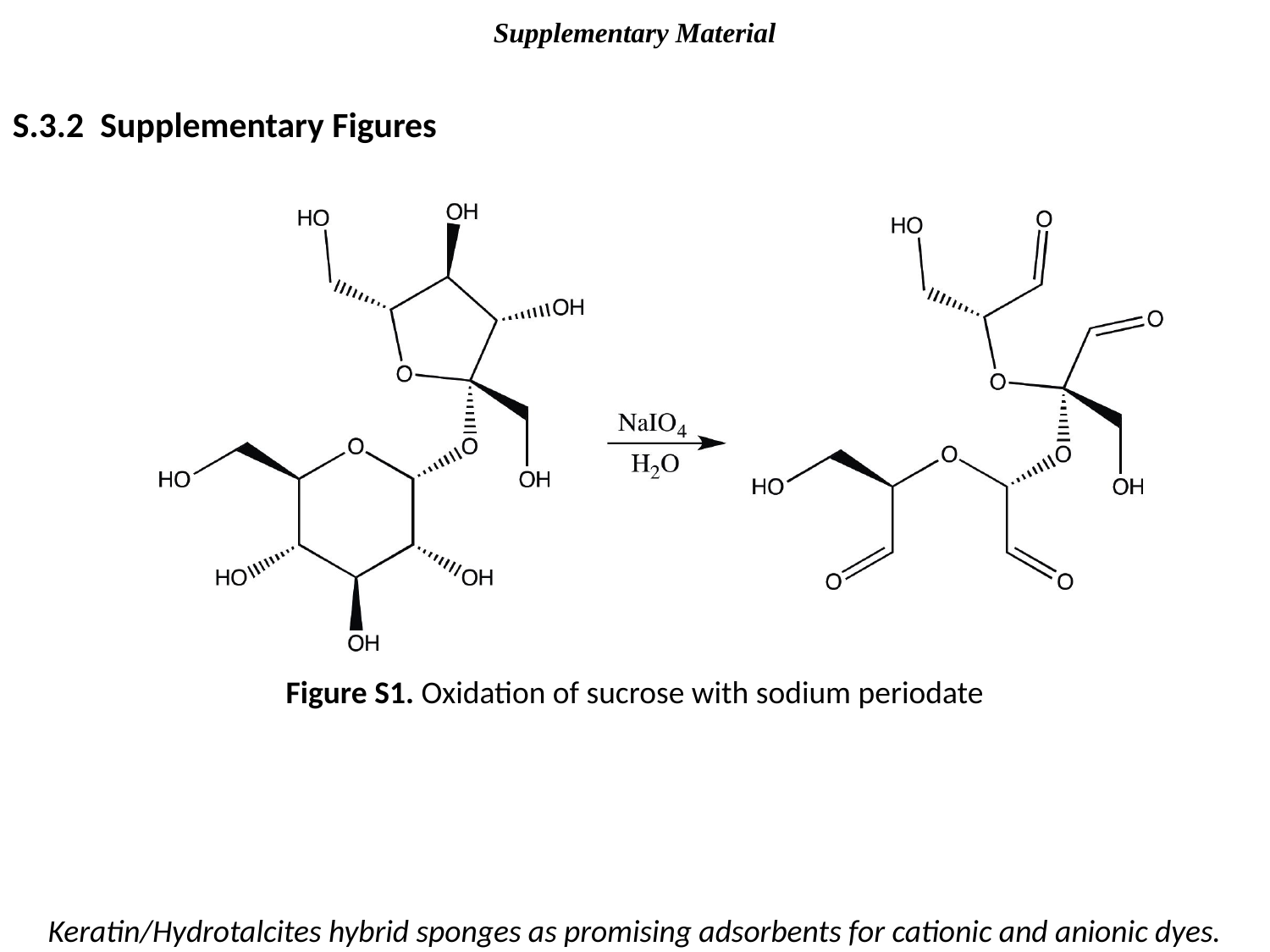

Supplementary Material
S.3.2 Supplementary Figures
Figure S1. Oxidation of sucrose with sodium periodate
Keratin/Hydrotalcites hybrid sponges as promising adsorbents for cationic and anionic dyes.

## Slide 5
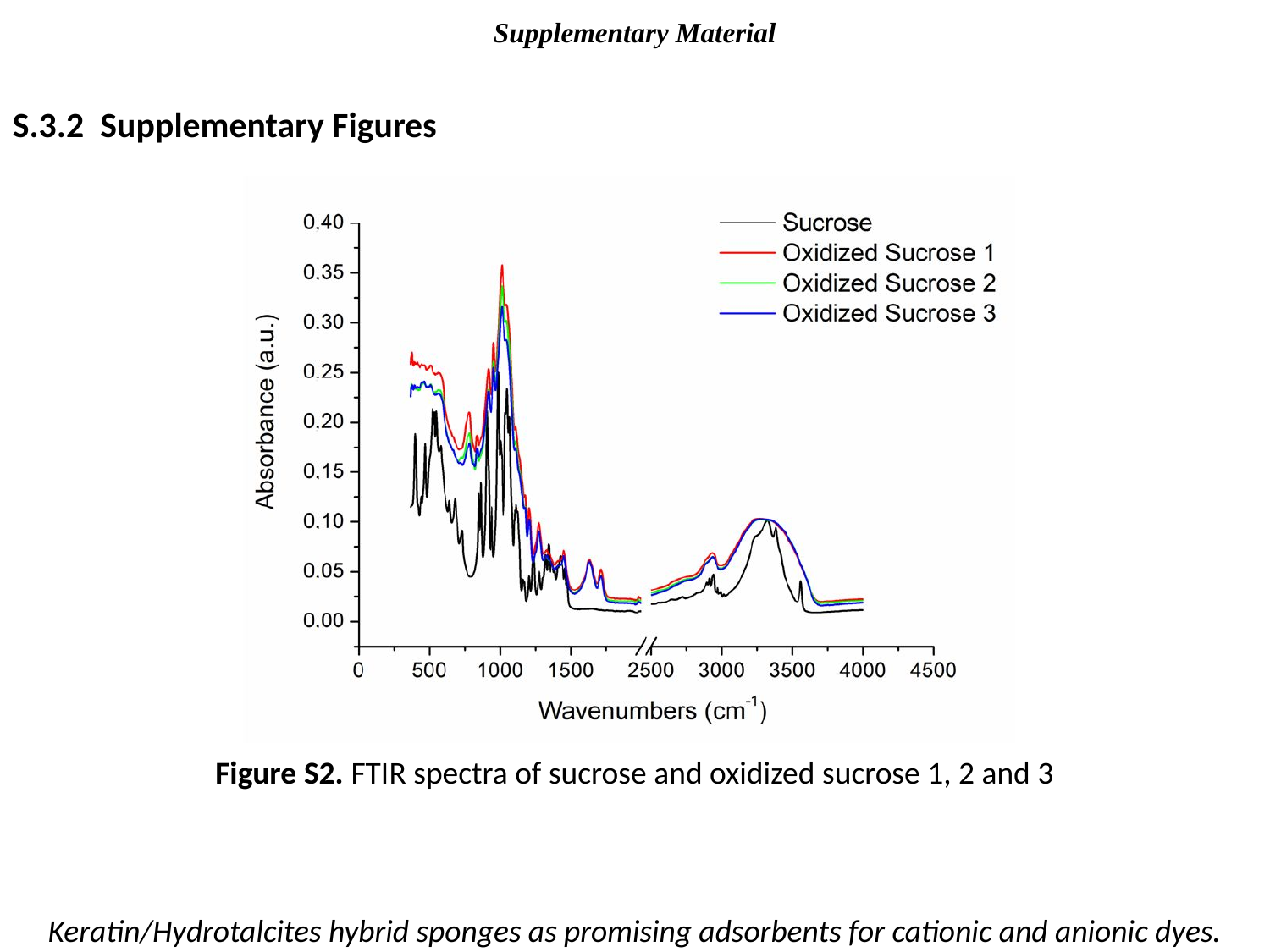

Supplementary Material
S.3.2 Supplementary Figures
Figure S2. FTIR spectra of sucrose and oxidized sucrose 1, 2 and 3
Keratin/Hydrotalcites hybrid sponges as promising adsorbents for cationic and anionic dyes.

## Slide 6
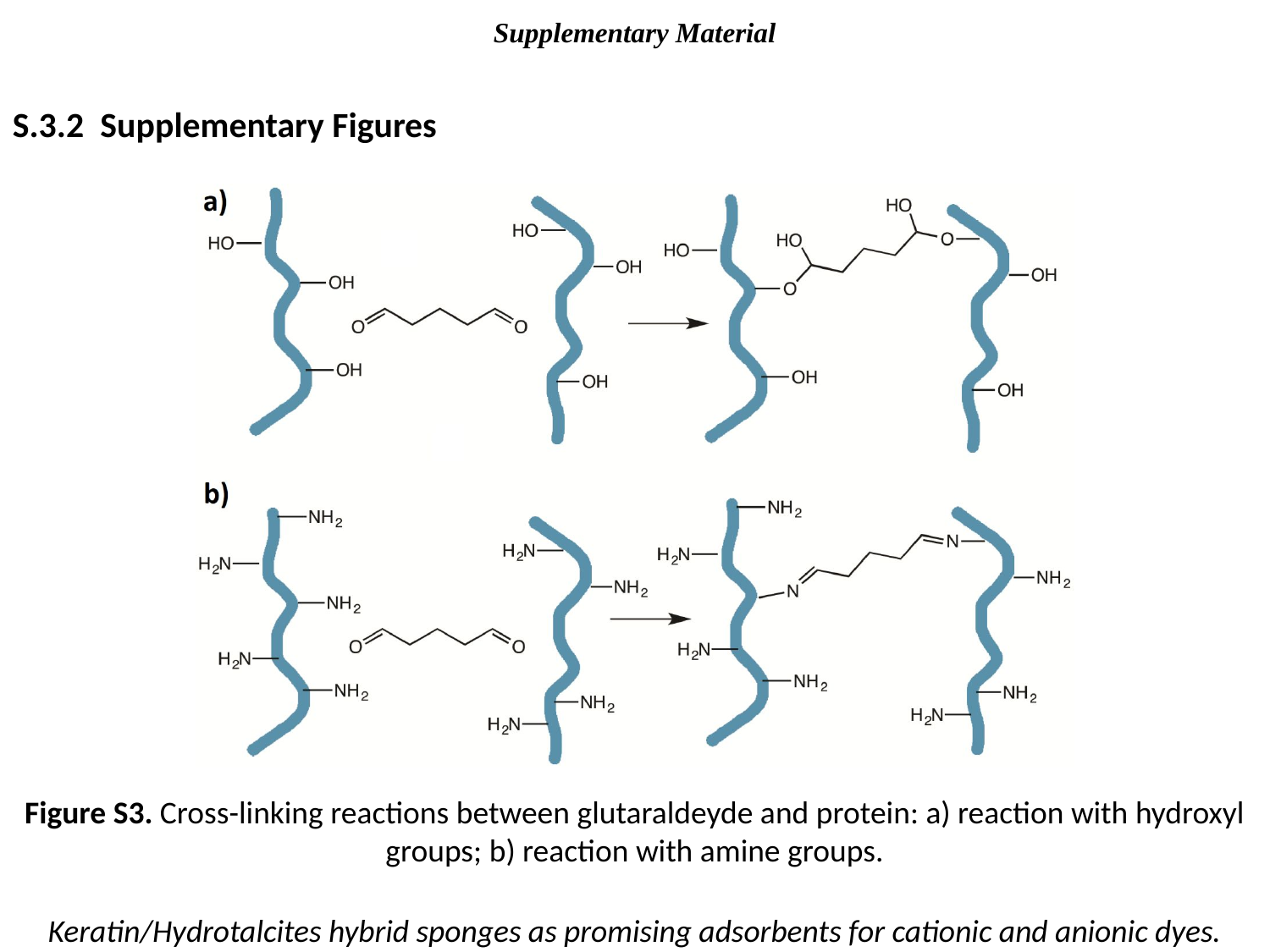

Supplementary Material
S.3.2 Supplementary Figures
Figure S3. Cross-linking reactions between glutaraldeyde and protein: a) reaction with hydroxyl groups; b) reaction with amine groups.
Keratin/Hydrotalcites hybrid sponges as promising adsorbents for cationic and anionic dyes.

## Slide 7
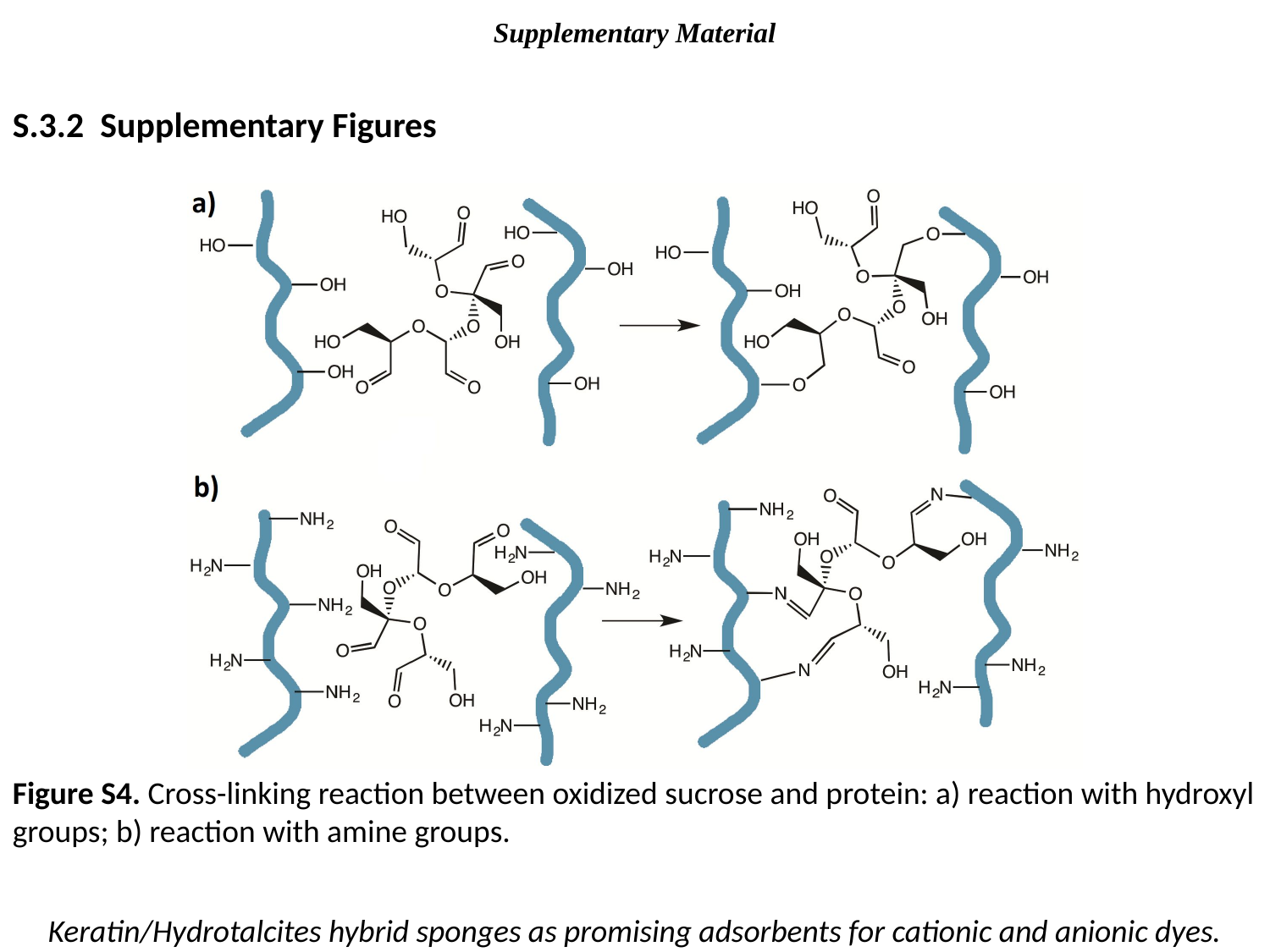

Supplementary Material
S.3.2 Supplementary Figures
Figure S4. Cross-linking reaction between oxidized sucrose and protein: a) reaction with hydroxyl groups; b) reaction with amine groups.
Keratin/Hydrotalcites hybrid sponges as promising adsorbents for cationic and anionic dyes.

## Slide 8
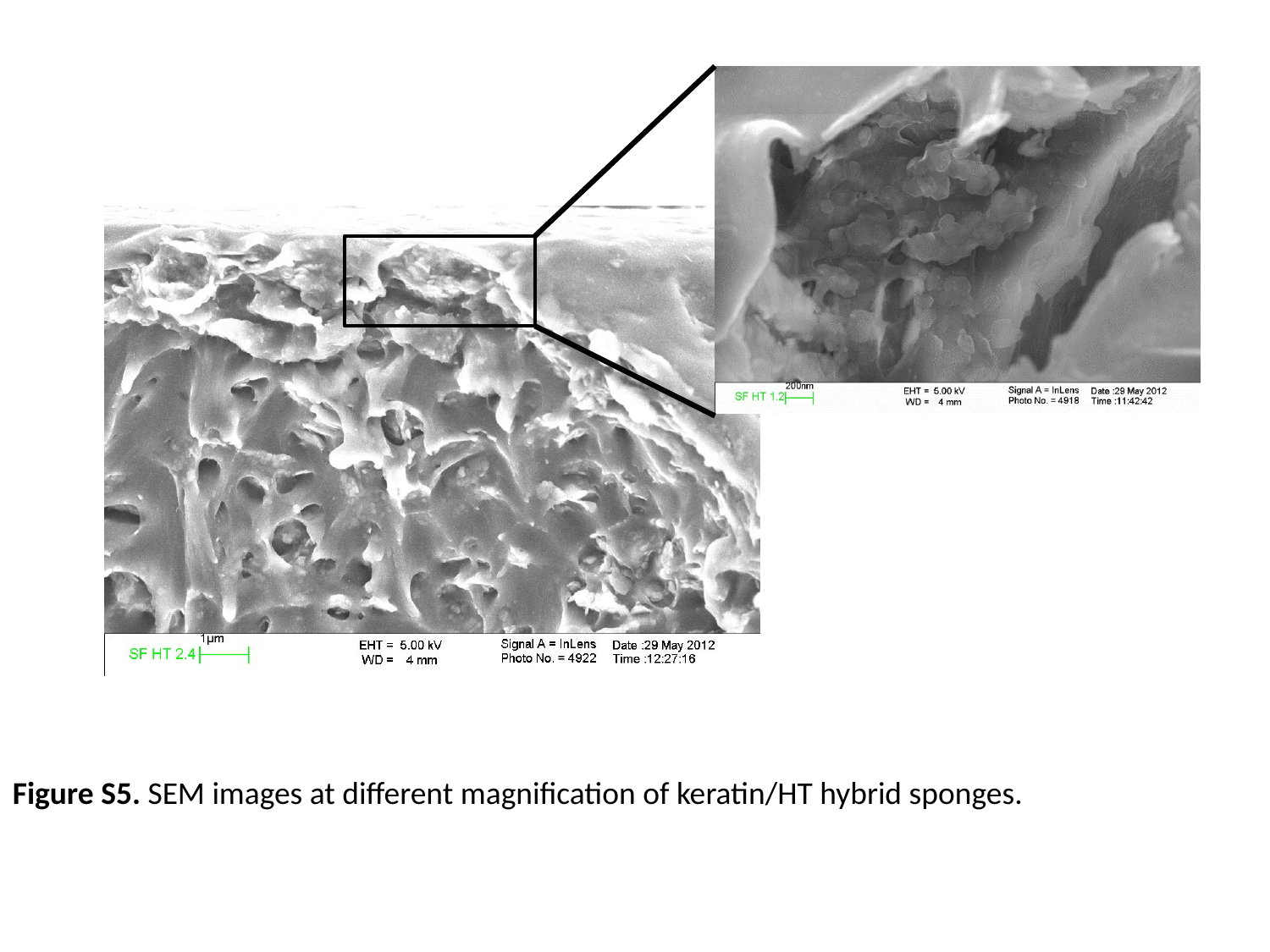

Figure S5. SEM images at different magnification of keratin/HT hybrid sponges.

## Slide 9
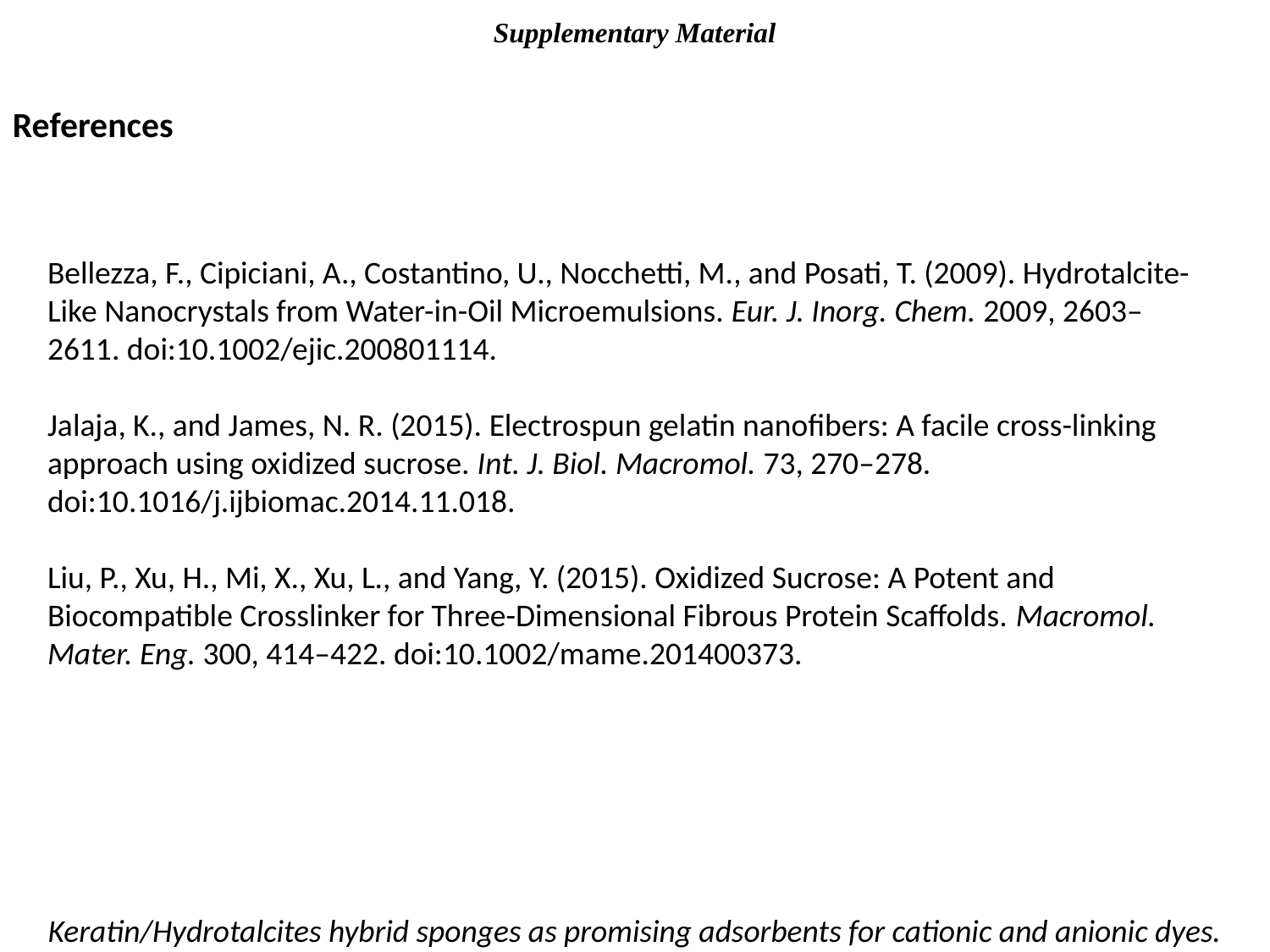

Supplementary Material
References
Bellezza, F., Cipiciani, A., Costantino, U., Nocchetti, M., and Posati, T. (2009). Hydrotalcite-Like Nanocrystals from Water-in-Oil Microemulsions. Eur. J. Inorg. Chem. 2009, 2603–2611. doi:10.1002/ejic.200801114.
Jalaja, K., and James, N. R. (2015). Electrospun gelatin nanofibers: A facile cross-linking approach using oxidized sucrose. Int. J. Biol. Macromol. 73, 270–278. doi:10.1016/j.ijbiomac.2014.11.018.
Liu, P., Xu, H., Mi, X., Xu, L., and Yang, Y. (2015). Oxidized Sucrose: A Potent and Biocompatible Crosslinker for Three-Dimensional Fibrous Protein Scaffolds. Macromol. Mater. Eng. 300, 414–422. doi:10.1002/mame.201400373.
Keratin/Hydrotalcites hybrid sponges as promising adsorbents for cationic and anionic dyes.
